# Infiltrating B-cell subtypes and associated hub genes in nasopharyngeal carcinoma identified from integrated single-cell, bulk RNA-sequencing, and immunohistochemical data

**DOI:** 10.1186/s41065-025-00414-7

**Published:** 2025-03-29

**Authors:** Fangyan Zhong, Junjun Chen, Tianzhu Lu, Lin Zhang, Zhiliang Liu, Chunhong Guan, Xiaopeng Xiong, Xiaochang Gong, Jingao Li

**Affiliations:** 1 Jiangxi Medical College, Nanchang University, Nanchang, China; 2 NHC Key Laboratory of Personalized Diagnosis and Treatment for Nasopharyngeal Carcinoma, Jiangxi Cancer Hospital, Nanchang, China; 3 Department of radiation oncology, Jiangxi cancer Hospital, Nanchang, China; 4 Department of Pharmacology, School of Pharmacy, Nanchang University, Nanchang, China; 5 Institute of Geriatrics, Jiangxi provincial People’s Hospital, First Affiliated Hospital of Nanchang Medical College, Nanchang, China; 6 Department of pathology, Jiangxi Cancer Hospital, Nanchang, China

**Keywords:** Nasopharyngeal carcinoma, Gene expression omnibus, B cells, Immune cell infiltration, Prognosis

## Abstract

**Background:**

Nasopharyngeal carcinoma (NPC) is associated with lymphocyte infiltration; however, the majority of research on NPC has focused on the role of T cells, with relatively little known about the roles of B cells and their subtypes. Therefore, we evaluated the prognostic value of CD20 + B cell density and B-cell subtypes along with their functional enrichment and hub genes in NPC.

**Methods:**

The prognostic value of CD20 + B-cell density for distant metastasis-free survival (DMFS), overall survival (OS), and progression-free survival (PFS) was explored by immunohistochemistry using multivariate analysis. Transcriptomic expression data from Gene Expression Omnibus (GEO) datasets were analyzed to identify B-cell subtypes and their functional enrichment in NPC tissues. Pseudotime trajectory analysis was performed to evaluate the B-cell differentiation trajectory and hub genes were identified using Cytoscape software.

**Results:**

Patients with NPC exhibiting a high infiltrating density of CD20^+^ B cells showed significantly better 5-year DMFS, OS, and PFS compared to those of patients with a low infiltrating density. Naïve B cells, switched memory B cells, exhausted B cells, and plasma cells were identified as key B-cell subtypes infiltrating NPC tumors, with naïve B cells showing the highest infiltration levels associated with a better prognosis. Naïve B cells were closely associated with immune pathways and the hub genes were typical markers for T and B cells.

**Conclusion:**

A high infiltrating density of B cells showed strong prognostic value in patients with NPC. Naïve B cells may play an important role in tumor immunity for NPC.

**Supplementary Information:**

The online version contains supplementary material available at 10.1186/s41065-025-00414-7.

## Introduction

Nasopharyngeal carcinoma (NPC) is characterized by a large number of lymphocytic infiltrates, leading to the term “lymphoepithelioma” to describe this malignancy [1]. More recently, it has been conceptualized as a ​"multidimensional spatiotemporal pathological ecosystem embodying the unity of ecology and evolution“ [2], emphasizing the dynamic interplay between tumor cells and their microenvironment across spatial and temporal dimensions. Many studies have shown that the density of tumor-infiltrating lymphocytes (TILs) is associated with the prognosis of patients with NPC [3]. However, different T-cell subtypes have distinct functions in the tumor immune microenvironment. TILs not only kill tumor cells and increase the function of cytotoxic CD8^+^ T cells but also induce tumor immunosuppression. Although the role of CD8^+^ T cells in the antitumor immune response in NPC has been validated, the role of B cells, as another important immune cell type, in this response remains unclear, warranting further exploration.

With deepening of the understanding of the tumor microenvironment (TME), the biological functions and antitumor immunity of B cells in tumors have gradually received increasing attention [4–6]. Many studies have found that the degree of infiltration (i.e., number and density) of B cells in TME is closely related to a good prognosis of patients with various types of cancer, such as lung cancer [7], colorectal cancer [8], hepatocellular carcinoma [9] and so on, which emerged as a more significant prognostic marker than CD8^+^ T cells. Moreover, the degree of B-cell infiltration in TME is closely related to the outcome of tumor immunotherapy. Therefore, further evaluation of the infiltration and function of B-cell subtypes in NPC can offer new prognostic markers and immunotherapy targets.

Recent advances in single-cell sequencing have enabled the identification of different subtypes of vital cells and their functions. In particular, in cancer research, tumors and their microenvironments have unique functions that cannot be explored by analysis of bulk RNA/DNA sequencing data. Recent studies on NPC have used single cell sequencing data to reveal the profiles of the TME [10–12]. However, these studies mainly focused on T-cell subtypes and did not specifically analyze the roles of B cells in the TME of NPC. Moreover, previous studies have used only single-cell sequencing data rather than integrated data from single-cell and bulk-RNA sequencing to explore the roles and clinical value of various immune cell subtypes in NPC.

In this study, to further explore the prognostic value of various tumor-infiltrating B-cell subtypes in NPC, we integrated single-cell and bulk-RNA sequencing data from public databases. Furthermore, we conducted pathway enrichment analysis for B-cell subtypes with prognostic significance and determined their hub genes to provide new insight into the TME changes associated with NPC and identify new treatment targets.

## Materials and methods

### Tissue samples and immunohistochemistry

Tissue samples from 217 patients with NPC diagnosed at Jiangxi Cancer Hospital between January 2014 and December 2015 were included for analysis. All the patients have accepted radical radiotherapy. All patients with NPC were reclassified according to the TNM stage classification scheme of the 8th edition of the American Joint Committee on Cancer staging manual. This study was approved by the Hospital Review Board of Jiangxi Cancer Hospital (No.2023ky102), Jiangxi, China. (More details in the supplementary materials and methods).

### Public data sources

Two NPC single-cell transcriptome datasets (GSE150430 and GSE162025) and two bulk-RNA transcriptome datasets (GSE102349 and GSE34573) were downloaded from the GEO database (http://www.ncbi.nlm.nih.gov/geo/). The GSE150430 dataset contained 15 primary NPC tumor tissues and one normal nasopharyngeal epithelial (NNE) tissue, with a total of 48,584 cells (46,001 cells from NPC tissues and 2583 cells from the NNE tissue). The GSE162025 dataset contained 10 NPC tissues and paired peripheral blood samples, with a total of 17,6447 cells. (More details in the supplementary materials and methods).

### Identification of immune cell subsets

Single cells were subjected to t-distributed stochastic neighbor embedding (t-SNE) [13] to identify five cell types (T cells, B cells, CD4^+^ T cells, CD8^+^T cells, and dendritic cells). Following standard Seurat procedures, four subsets of B cells (naïve B cells, switched memory B cells, exhausted B cells, and plasma cells) were identified from 19,154 B cells. (More details in the supplementary materials and methods).

### Gene set enrichment of B-cell subsets

Hallmark genes associated with B-cell subsets in NPC were evaluated using gene set enrichment analysis(GSEA) [14], single-sample gene set enrichment analysis (ssGSEA) [15], and gene set variation analyses(GSVA) [16]. (More details in the supplementary materials and methods).

### Differentially expressed genes according to B-cell infiltration levels

Differential gene expression analysis of samples with high and low levels of infiltrating B cells (based on the median value of infiltration) was conducted in the GSE102349 dataset using the R package “limma” [17] according to thresholds of p < 0.05 and|log2 fold change| > 1. The differentially expressed genes (DEGs) were visualized with heatmaps and volcano plots using the R package “pheatmap” and “EnhancedVolcano,” respectively.

### Pathway and functional enrichment analysis

Pathway and functional enrichment analyses of the DEGs between high- and low-infiltrating B-cell groups were performed with Kyoko Encyclopedia of Genes and Genomes (KEGG) and Gene Ontology (GO) databases using the R package “ClusterProfiler” (version 4.0.5) [18]. A threshold of *p* < 0.05 was used to screen for significantly enriched pathways and functions.

### Cell–cell interactions and protein–protein interaction (PPI) network

We used CellphoneDB [19] to identify interactive pairs with default parameters and to predict receptor–ligand interactions. In addition, we used the STRING database to conduct PPI analysis of the DEGs. (More details in the supplementary materials and methods).

### Statistical analysis

Pearson χ2 test or Fisher’s test was used to compare the basic clinical characteristics of patients in different groups. Kaplan–Meier survival analyses were used to estimate the DMFS, OS, PFS and Recurrence-free survival (RFS) with log-rank test for comparison of survival curves. Multivariate analyses with Cox proportional hazard methods were used to estimate the risk of death. (More details in the supplementary materials and methods).

## Results

### Association of CD20^+^ B-cell infiltration with prognosis in NPC

**Fig. 1:**
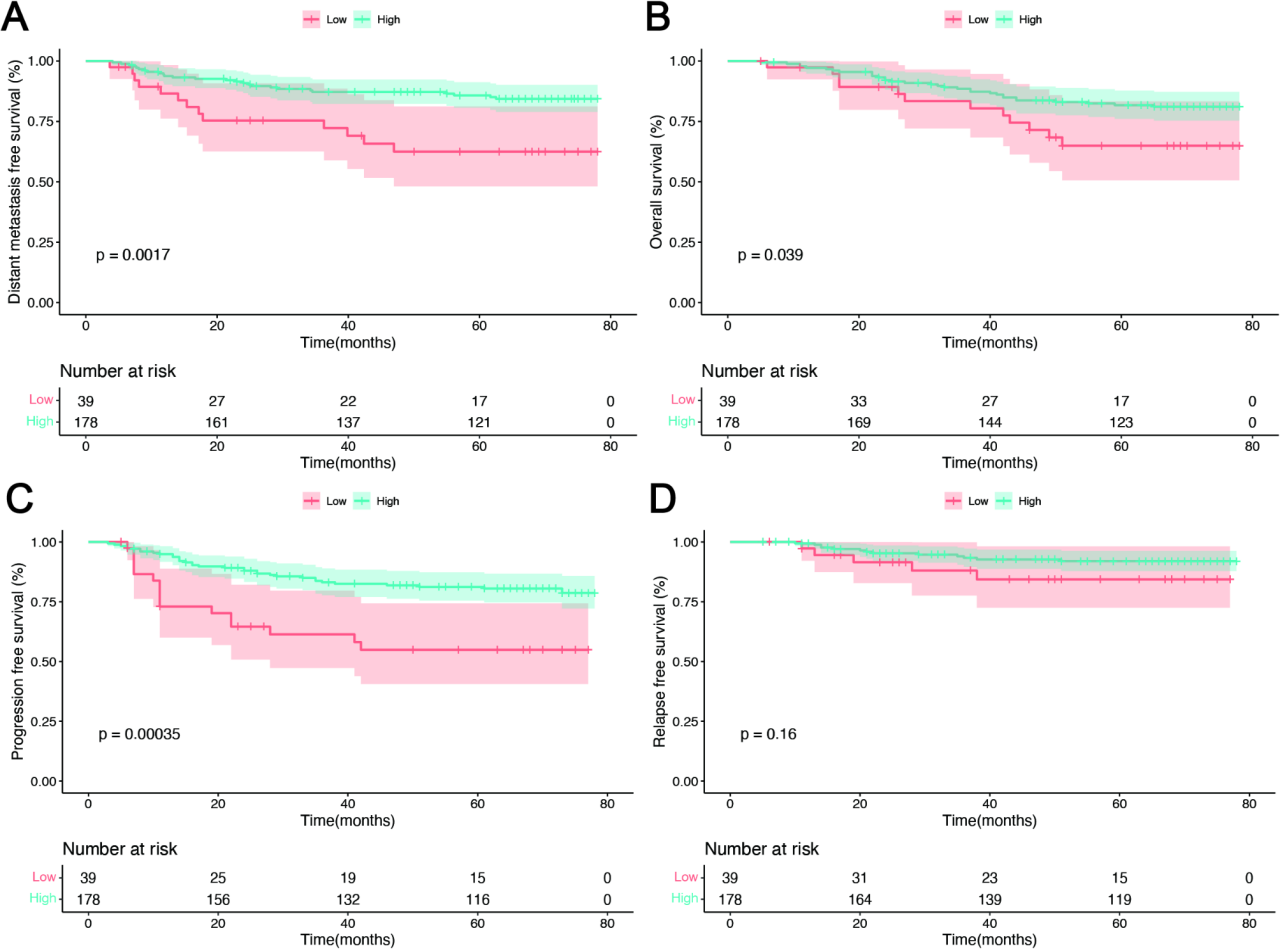
Survival curves of patients with NPC according to different levels of B-cell infiltration. (**A**) Distant metastasis-free survival. (**B**) Overall survival. (**C**) Progression-free survival. (**D**) Recurrence-free survival

Tissues of a total of 217 patients with NPC (69 female patients and 148 male patients; median age of 48 years, interquartile range 38–56 years) were included in the immunohistochemistry analysis to detect CD20^+^ B-cell density (Representative B cell IHC staining images are shown in Fig.S1A-D). The detailed clinicopathological features of the patients are shown in Table S1. According to the best cutoff value of the infiltrating density of CD20^+^ B cells in the tumor area (601.9 cells/mm^2^), patients were classified into high- and low-infiltration groups. The Kaplan–Meier survival analysis showed that patients with a high infiltrating density of CD20^+^ B cells had better 5-year DMFS (87% vs. 66.7%, *p* = 0.0017), 5-year OS (82.6% vs. 69.2%, *p* = 0.039), and 5-year PFS (82.0% vs. 59.0%, *p* = 0.00035) than those of patients in the low infiltration group (Fig. 1A–C). However, RFS was not significantly different between the two groups (84.4% vs. 92%, *p* = 0.16; Fig. 1D). After adjusting for gender, age, T classification, N classification, and Epstein–Bar virus DNA status, multivariate analysis showed that CD20^+^ B-cell infiltrating density was an independent prognostic factor of DMFS, OS and PFS for patients with NPC (Table S2).

### B-cell profiles in normal nasopharyngeal epithelial and NPC tissues

Considering that the density of B cells was significantly correlated with the prognosis of patients with NPC, we further explored the value of different B-cell subtypes using public single-cell and bulk-RNA sequencing datasets. Screening of the GSE150430 dataset (Fig. S2A–C) identified 31 cell clusters with different patients clustered in different cell clusters, indicating tumor heterogeneity among the 15 patients with NPC (Fig. S3A-B). To compare the differences in the clusters of various immune cells between NPC and normal nasopharyngeal epithelial (NNE) tissues, we extracted 2555 immune cells from the one NNE tissue sample and 45,455 immune cells from the 15 primary NPC tissue samples (Fig. S3C-D). The NPC tissues generally had higher proportions of dendritic cells, CD4^+^ T cells, and CD8^+^ T cells than NNE tissues (Fig. S3E), whereas only the proportion of B cells was higher in the NNE tissue than in NPC tissues (Fig. S3F).

**Fig. 2:**
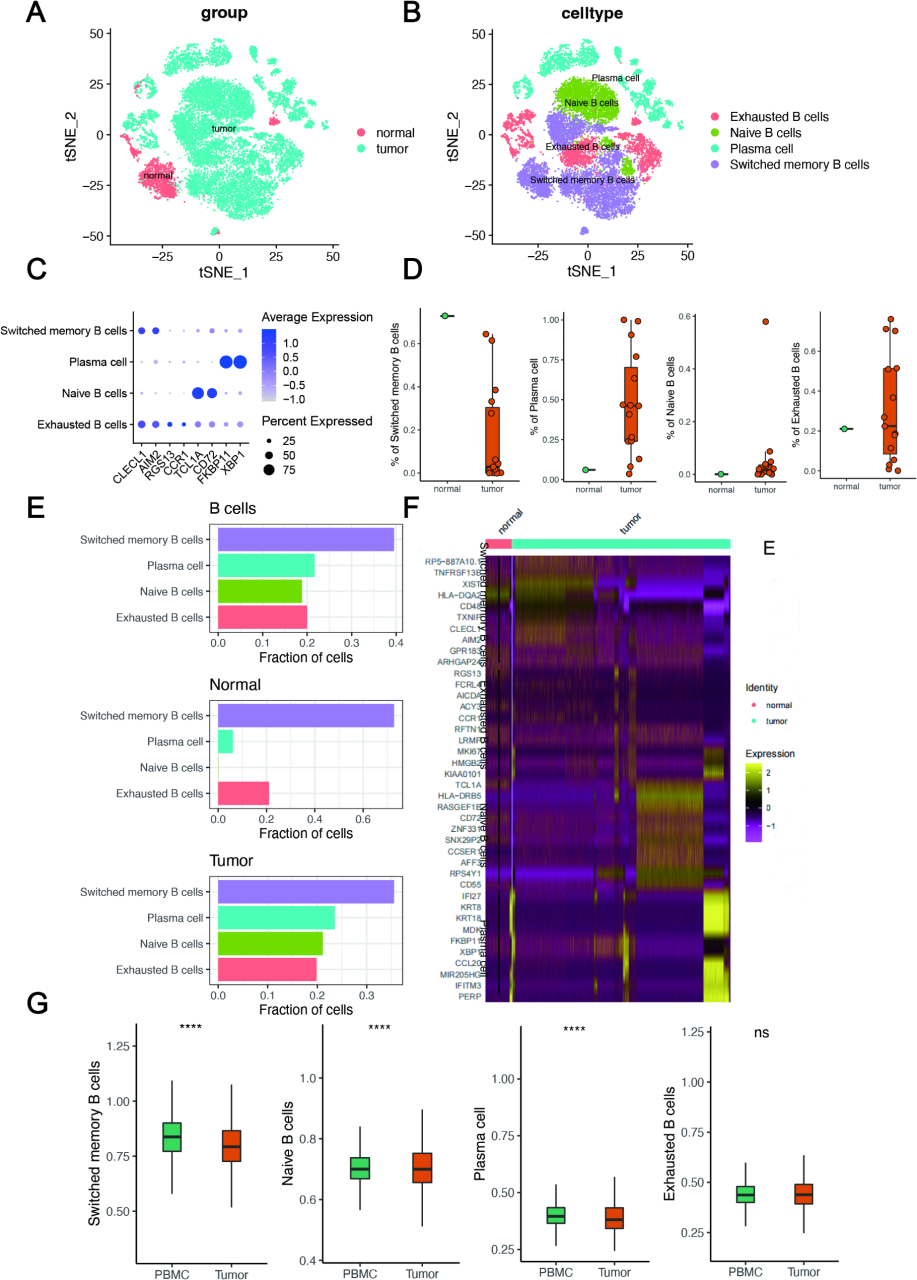
Different subtypes of B cells in normal nasopharyngeal epithelial (NNE) and NPC tissues. (**A**) t-distributed stochastic neighbor embedding (t-SNE) plot of B cells. (**B**) t-SNE plot for four subtypes of B cells. (**C**) Expression of different markers of B-cell subtypes. (**D**) Proportions of different B-cell subtypes between NNE and NPC tissues. (**E**) Fractions of the four B-cell subtypes relative to total B-cell counts. (**F**) Expression levels of markers of the four B-cell subtypes presented as a heatmap. (**G**) Boxplots showing differences of B-cell subsets in NPC tissues and paired peripheral blood samples

To further explore the diversity of B cells, we analyzed 2064 and 17,090 single cells using RNA-sequencing data from NNE and NPC tissues, respectively. Based on their transcriptional characteristics, B cells were classified as naïve B cells, switched memory B cells, plasma cells, or exhausted B cells (Fig. 2A-B). Switched memory B cells highly expressed *CLECL1* and *AIM2*, the marker genes of naïve B cells were *TCL1A* and *CD72*, those of plasma cells were *FKBP11* and *XBP1*, and those of exhausted B cells were *RGS13* and *CCR1* (Fig. 2C). Further analyses showed that plasma cells, naïve B cells, and exhausted B cells highly infiltrated the tumor tissues, whereas switched memory B cells highly infiltrated NNE tissues (Fig. 2D). In the NNE tissue, there were almost no naïve B cells, whereas the number of memory B cells was higher in the NNE tissue than in NPC tissues (Fig. 2E). We also analyzed the expression patterns of markers for all four B-cell subtypes across both NPC and NNE tissues using a heatmap (Fig. 2F). In addition, we explored the differences in B cells between NPC tissues and paired peripheral blood samples in the GSE162025 dataset. Compared with NPC tissues, switched memory B cells showed greater accumulation in the peripheral blood (Fig. 2G), whereas naïve B cells showed the greater accumulation in the tumor tissue (Fig. 2G).

### Prognostic value of various B-cell subtypes in NPC

**Fig. 3:**
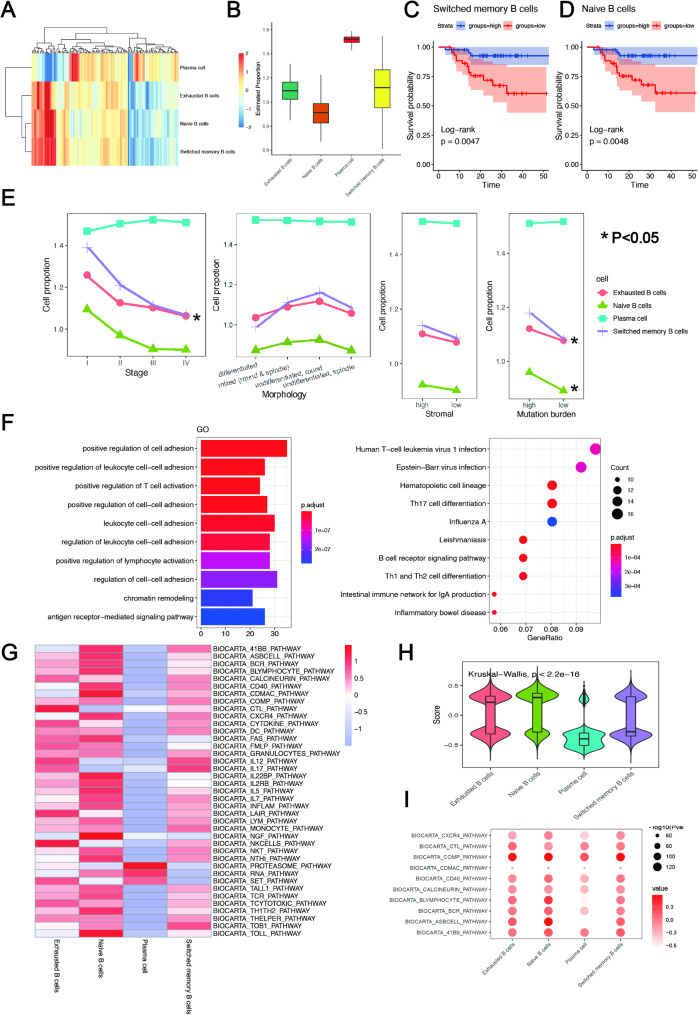
Proportion of different B-cell subtypes and their association with prognosis in NPC. (**A**-**B**) Heatmap (**A**) and boxplot (**B**) showing the proportion of different B-cell subtypes in bulk RNA-sequencing data. (**C**-**D**) Survival curves of switched memory B cells and naïve B cells. ((**E**) Correlation between the proportion of different B cell subtypes in total cells and different clinical features. (**F**) Gene Ontology and Kyoto Encyclopedia of Genes and Genomes enrichment of differentially expressed gene in naïve B cells. (**G**) Immune checkpoint pathway activity of B-cell subtypes. (**H**) Distribution of immune pathway activity in different B-cell subtypes. (**I**) The top 10 immune checkpoint pathways significantly enriched in all B-cell subtypes. The node size represents the–log10 p-value. Color intensity is based on the enrichment score

The GSE102349 dataset, which included 113 NPC samples, was deconvolved using ssGSEA according to the characteristic genes of the four B-cell subclusters. Naïve B cells switched memory B cells, plasma cells, and exhausted B cells were detected in the NPC samples, with a relatively higher proportion of plasma cells detected (Fig. 3A, B). Survival analyses showed that patients with high infiltration of naïve B cells had better OS than those with low infiltration (Fig. 3C). Switched memory B cells had a similar situation (Fig. 3D). The infiltration of plasma cells or exhausted B cells had no significant correlation with OS. In addition, the proportion of plasma cells increased with clinical stage, while the other three types of B cells showed the opposite pattern, especially switched memory B cells (Fig. 3E). Naïve B cells, exhausted B cells, and switched memory B cells were the most common pathological features of the undifferentiated round cells (Fig. 3E). All B cells showed a higher proportion of samples with high stromal scores (Fig. 3E). In addition, samples with a high mutation burden had a significantly higher proportion of naïve and memory B cells, similar to exhausted B cells. In contrast, plasma cells were observed in a higher proportion of patients with a low mutation burden than a high mutation burden (Fig. 3E).

Next, to explore the functions of different B-cell subtypes, we performed GO and KEGG enrichment analyses for DEGs within each subtype. Naïve B cells, switched memory B cells, plasma cells, and exhausted B cells had 413, 91, 1256, and 135 significantly upregulated genes, respectively. GO and KEGG analyses showed that DEGs in naïve B cells were enriched in the T cell activation, B-cell receptor signaling pathway, and other functions and pathways that promote the immune response (Fig. 3F). We further explored the prognostic value of signatures in T cell activation and B-cell receptor signaling pathway, respectively. NPC patients with high infiltration of T cell activation pathway had better OS than those with low infiltration (*P* = 0.018, Fig. S4A and Table S3). While NPC patients with high B cell receptor signaling pathway had better OS than those with low infiltration, while the prognostic value of the B cell receptor signaling pathway was a trend significance in NPC (*P* = 0.062, Fig. S4B and Table S3).

DEGs in the switched memory B cells were enriched in B-cell activation and immune cell proliferation based on GO enrichment (Fig. S5A, B). DEGs in plasma cells were enriched in metabolism-related functions and pathways such as oxidative phosphorylation (Fig. S1C, D). Exhausted B cells were significantly enriched with mitosis, chromatin remodeling, and other functions, but no enriched KEGG pathway was found (Fig. S5E). We further analyzed the associated dataset of metabolic pathways, hallmarks, and immune checkpoint pathways from the MsigDB database and calculated the activity of these pathways in B-cell subsets using the R package “GSVA.” The results showed that the immune checkpoint pathway was the most significantly enriched pathway in naïve B cells (Fig. 3G, H). The B-cell receptor signaling pathway was significantly enriched in both exhausted and naïve B cells (Fig. 3I). Plasma cells exhibited more metabolism-related functions (Fig. S5F-K).

### Cell differentiation trajectory analysis

**Fig. 4:**
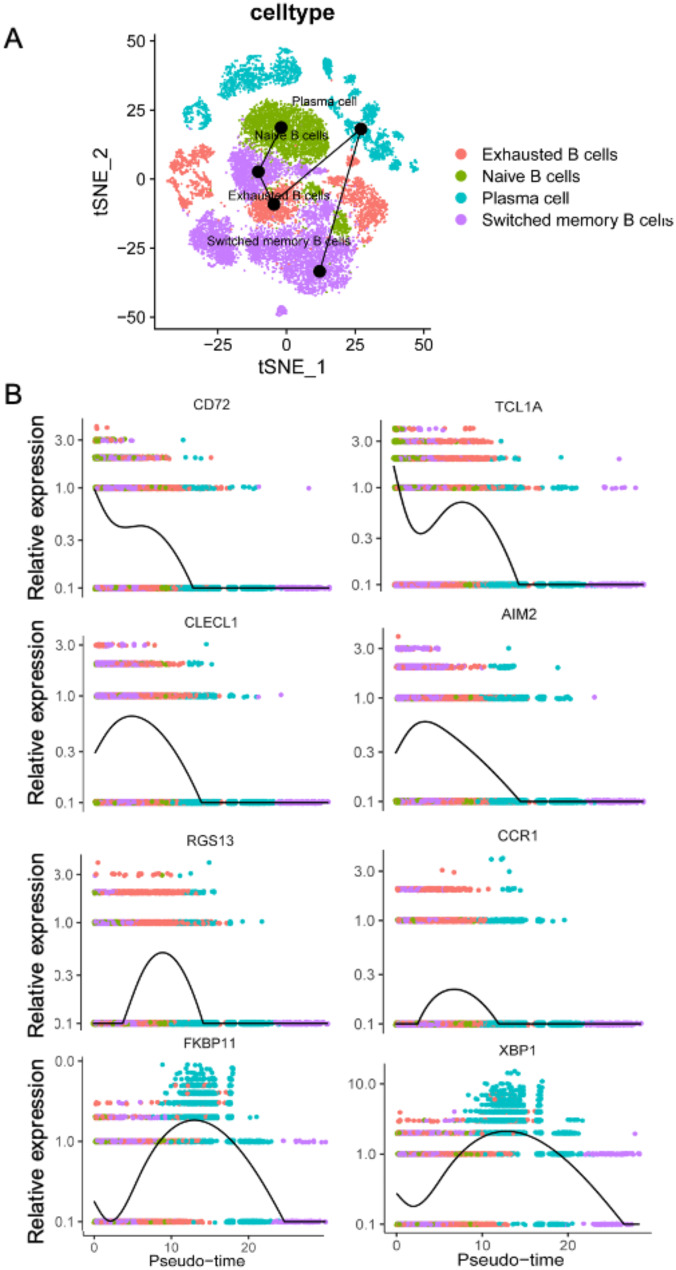
Cell trajectory analysis and changes of marker genes. (**A**) t-distributed stochastic neighbor embedding plots for cell differentiation trajectory analysis. (**B**) Changes in expression profiles of marker genes for different B-cell subtypes

Pseudo-time trajectory analysis was used to explore the B-cell differentiation procession by the R package “monocle.” Naïve and switched memory B cells tended to transform into exhausted B and plasma cells (Fig. 4A). During the transformation of naïve and switched memory B cells into exhausted B cells, the gene expression of naïve B cell markers (*CD72* and *TCL1A*) and switched memory B cell markers (*CLECL1* and *AIM2*) was gradually downregulated (Fig. 4B), whereas the expression of markers of exhausted B cells (*RGS13* and *CCR1*) and plasma cells (*FKBP11* and *XBP1*) was upregulated (Fig. 4B). Based on the heterogeneity of different B-cell subtypes, we analyzed their communication networks to identify key ligand–receptor pairs and cell subtypes that dominate the interactions. We found that plasma cells played a dominant role in this interaction (Fig. S6A, B). Among them, the top five ligand–receptor pairs were the a10b1, a11b1, a1b1, aVb3, and a2b1 complexes (Fig. S6C). In addition, several intracellular interaction pairs were identified in the plasma cells, and HLA-DPA1_TNFSF9 interaction relationships were significant between most cells (Fig. S6D).

### Prognostic and treatment markers among hub genes of Naïve B cells

**Fig. 5:**
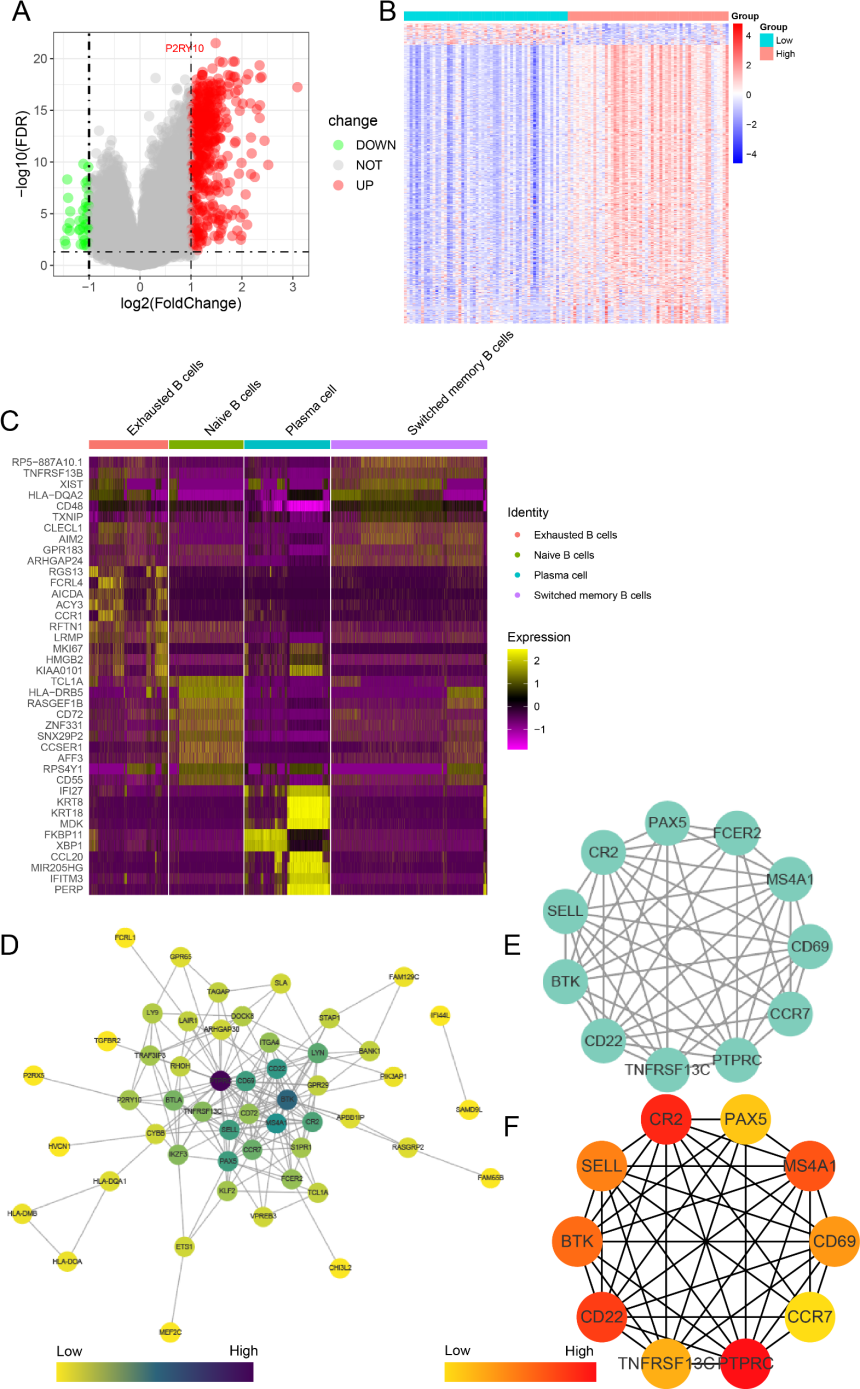
Identification of hub genes. (**A**, **B**) Differentially expressed genes (DEGs) between patients with a high and low proportion of infiltrating naïve B cells. The gene symbol is labeled; DEGs were selected according to a p-value < 0.05 and|log2 fold change| > 1. Red represents upregulated gens and green represents downregulated genes. (**C**) DEGs between the four B-cell subtypes from the single-cell RNA-sequencing dataset. (**D**) Protein–protein interaction network showing the co-expression patterns of DEGs identified in the bulk-RNA dataset and single-cell RNA-sequencing dataset. The intensity of the color represents the magnitude of the degree of correlation. (**E**) Subnetwork from the co-expressed genes. (**F**) Degree of hub genes

Given the finding that a high proportion of naïve B cells contributes to a better prognosis in patients with NPC and that these cells were associated with DEGs that are significantly enriched in functions of the immune response, we identified the hub genes among these DEGs and further analyzed their functions. A total of 470 upregulated and 36 downregulated genes were identified in samples with high and low proportions of naïve B cells in the GSE102349 dataset (Fig. 5A, B). A total of 413 genes that were highly expressed in naïve B cells were identified by analyzing the differences in the four B-cell subtypes in the single-cell RNA-sequencing dataset (Fig. 5C). Co-expression analyses identified 64 intersecting genes from the two DEG clusters. PPI network analysis based on these 64 genes showed that *CCR7*,* CD22*,* PAX5*,* TNFRSF13C*,* CR2*,* CD69*,* BTK*,* SELL*,* PTPRC*, and *MS4A1* (*CD20*) were hub genes (Fig. 5D–F).

We then grouped samples based on the median expression level of each hub gene and performed Kaplan–Meier survival analysis to explore their prognostic value. Kaplan–Meier analyses found that *CCR7*,* CD22*,* TNFRSF13C*,* CR2*,* SELL*, *PTPRC*, and *MS4A1* were advantage prognosticator of OS in NPC, respectively (Fig. S7A-G). In addition, we found that the expression levels of all hub genes decreased with the development of the pathological stage of NPC. *CCR7*,* CD22*,* TNFRSF13C*,* CR2*,* CD69*,* PAX5*, and *MS4A1* expression levels were significantly lower in advanced stages, with the highest expression level found in patients with stage I NPC (Fig. S8A). Further, GSE34573 dataset, including four normal samples and 16 tumor samples, showed that both *CD69* and *SELL* were over-expression in NPC (Fig. S8B).

### Association of IgD cells infiltration with prognosis in NPC

**Fig. 6:**
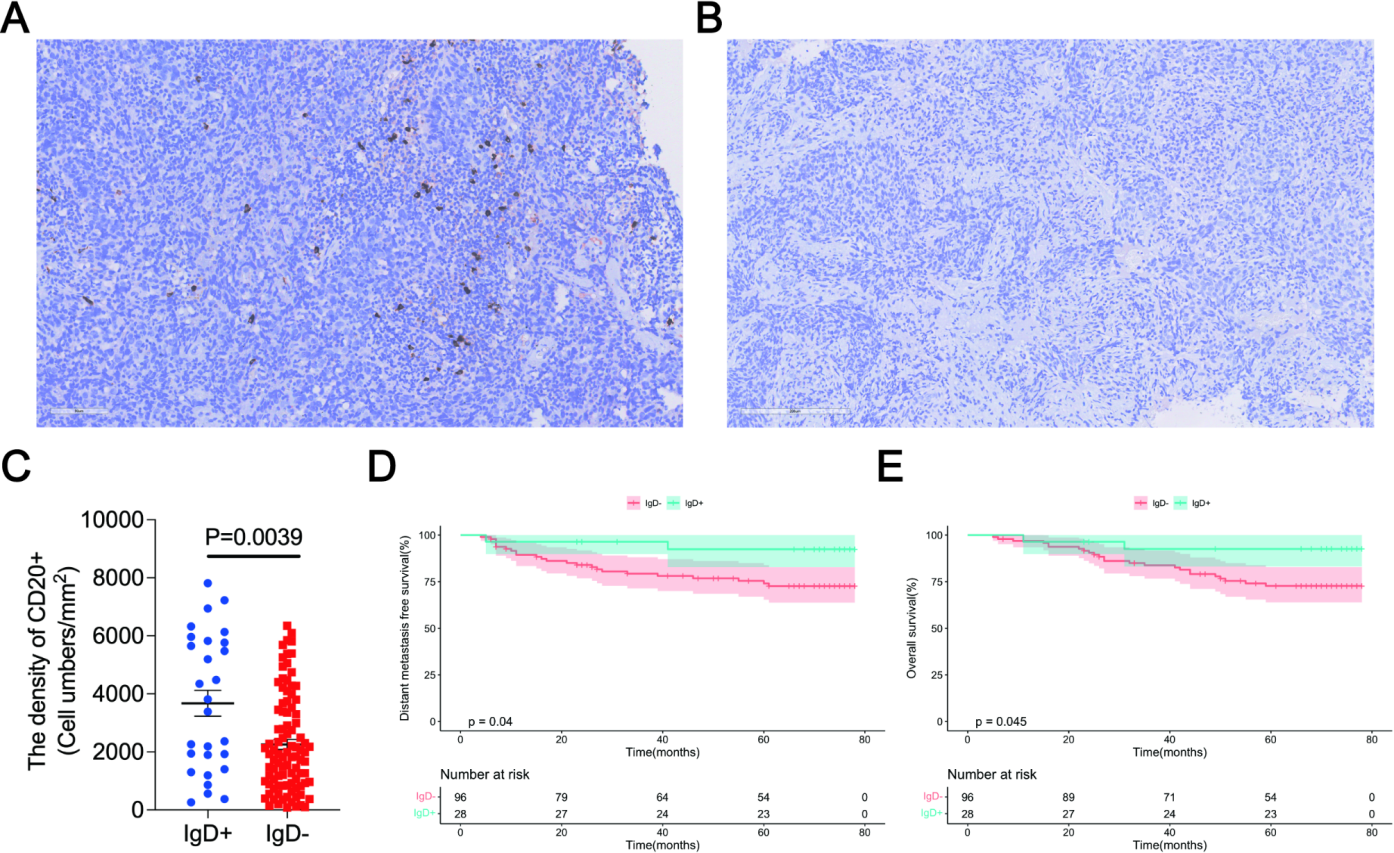
The NPC patients with IgD + cells shows the better survival. (**A**) IgD + cells IHC staining images. (**B**) IgD- cells IHC staining images. (**C**) IgD + patients with NPC has the higher CD20 + density than the IgD- patients. (**D**) Distant metastasis-free survival in NPC patients with IgD positive or IgD negative. (**E**) Overall survival in NPC patients with IgD positive or IgD negative

In order to analyzes the prognostic value of infilitrating naive B cell, the IgD was detected by IHC in remaining tissue after detection of CD20. One hundred twenty four patients with NPC, 30 female patients and 94 male patients, were included analyses. Representative IHC staining images are shown in Fig. 6A and B. IgD + NPC patients has the higher CD20 + density than the IgD- patients (Fig. 6C). The Kaplan–Meier survival analysis showed that patients with IgD + cells had better 5-year DMFS (92.4% vs. 74.0%, *p* = 0.04) and 5-year OS (92.6% vs. 72.7%, *p* = 0.045) than those of patients in the IgD- group (Fig. 6D, E).

## Discussion

The biological role of B cells in the TME has attracted increasing attention in recent years, and many studies have confirmed the prognostic value of the degree of B-cell infiltration in tumors [7]. In this study, the prognostic value and biological functions of B cells and their subtypes in NPC tissues were systematically analyzed using single-cell transcriptome, transcriptome, and immunohistochemistry data. We found that patients with a high density of infiltrating CD20^+^ B cells had better OS than those with a low density of these cells in the tumor tissue. Analyses of single-cell sequencing data of the GSE150430 GEO dataset revealed that the densities of naïve B cells, plasma cells, and exhausted B cells in NPC tissues were higher than those in the NNE tissue. Further analyses showed that patients with high levels of naïve B cell infiltration had better OS than those with low infiltration levels. Naïve B cells were mainly enriched in T-cell activation, antigen receptor-mediated signaling pathways, and B-cell receptor signaling pathways. Hub genes associated with naïve B cells were closely related to pathways associated with T and B cells. The NPC patients with IgD + cells show the better DMFS and OS than the IgD- patients.

In recent decades, B cells have been shown to play an immune-suppressing role in cancer treatment. This is because B cells can not only induce antibody-mediated immune suppression but also secrete pro-tumor genic factors [20]. However, with deeper research on the correlation between B cells and antitumor immunity, accumulating evidence shows that B cells can also promote antitumor immunity. In addition to enhancing the function of T helper cells, B cells can also recognize antigens, differentiate, and directly or produce antibodies to kill tumor cells [21–23]. Those anti-tumor effects are mainly caused by the pathways of B cells, such as CCL19, -21/CCR7 axis and CXCL13/CXCR5 axis [23]. It is worth mentioning that the crosstalk between B cells and other immune cells, especially T cells, has been gradually paid more attention by researchers. B cells loaded with antigenic peptides interact with CD4 + and CD8 + T cells, causing them to be activated and lead to Th1 and Th2 type immune responses [24, 25]. What’s more, B cells can combine with T cells and other immune cells to form a cluster distribution state, which is called tertiary lymphoid structure (TLS), and several studies have confirmed that TLS has good prognostic and predictive value [21, 26]. Indeed, the phenomenon of co-localization of T cells and B cells and TLS also observed in our sample (Fig. S9A-C). In addition, researchers had found high expression of TLS can improve the prognosis in NPC patients [27]. Meanwhile, several studies have shown that high B-cell infiltration was a better prognostic factor for HPV-associated squamous cell carcinomas [28], non-small cell lung cancer [29], esophageal cancer [30], hepatocellular carcinoma [5, 9], gastric cancer [31], and breast cancer [32]. Although previous studies have not reported the prognostic value of CD20^+^ B cells in NPC, our findings are consistent with those of other tumors. Our study also showed that the density of infiltrating CD20^+^ B cells was an independent prognostic factor.

Although B cells have shown significant prognostic value for patients with tumors, the B-cell subtypes that play a major role in promoting the tumor immune response have not been fully elucidated. Our study found that B cells in NPC can be divided into four common subtypes: naïve B cells, switched memory B cells, plasma cells, and exhausted B cells, which is consistent with other studies [29, 33]. We found that patients with NPC with high infiltration of naïve B cells or switched memory B cells had a better prognosis than that of patients with low infiltration levels of these B-cell subtypes. Furthermore, the number of naïve B cells declined with increasing NPC stage. This result was also consistent with previous studies in non-small-lung cancer [29], triple-negative breast cancer [33] and hepatocellular carcinoma [9].

In addition, higher infiltrating naïve B cell levels were significantly correlated with higher expression of both programmed death-1 (PD-1)/programmed death-ligand 1 (PD-L1) and the tumor mutation burden in patients with non-small cell lung cancer [29], which is consistent with our finding that the DEGs of naïve B cells were mainly enriched in immune-associated pathways, implying that patients with NPC showing high levels of naïve B cell infiltration might benefit from anti-PD-1/PD-L1-based immunotherapy [29, 33]. Several studies have also confirmed that among patients receiving immunotherapy, those with a high degree of B-cell infiltration can benefit from immunotherapy [7, 34, 35]. However, the prognostic value of B-cell infiltration also depends on the degree of CD8^+^ T cell infiltration. The present KEGG enrichment analysis showed that memory B cells were not enriched in immune-related pathways in NPC. In addition, memory B cells showed higher infiltration in NNE than in NPC tissues. This may be because memory B cells, which are the downstream molecules of antitumor immunity, are not involved in early tumor immunity. It is interesting to note that existing studies have also explored the complex heterogeneity of B-cell subsets and their dual roles within the tumor microenvironment. CD86 + memory B cells can promote tumor proliferation and immune evasion via the CD46-JAG1 signaling pathway while also being associated with antitumor immune responses. Lin et al.‘s study established a CD86 + memory B cell-based risk scoring model incorporating 14 differentially expressed genes, which effectively predicts patient prognosis. Furthermore, targeting the CD46-JAG1 pathway emerged as a potential novel immunotherapy strategy. These findings not only provide new insights for our subsequent research but also inspire us to conduct in-depth explorations of the heterogeneity within different B-cell subsets in the future [36].

The current study revealed that high infiltration of naive B cells and memory B cells in NPC tissues was significantly associated with favorable survival outcomes, whereas plasma cells and exhausted B cells did not exhibit similar prognostic value. This phenomenon may be closely related to the distinct functional roles of these B-cell subsets: naive B cells activate CD4⁺/CD8⁺ T cells through antigen presentation and collaborate with T cells to form TLS [21, 26], serving as immune hubs within non-lymphoid tissues to enhance antitumor immunity. Memory B cells, meanwhile, maintain long-term tumor surveillance through antibody secretion or immune memory functions [23], and their plasticity enables rapid reactivation as naive B cells upon tumor antigen recognition to restart immune responses. Plasma cells, although involved in antibody production, may be functionally suppressed by the tumor microenvironment and limited by their own cell numbers [37]. Exhausted B cells, characterized by high expression of inhibitory molecules such as PD-1, contribute to immune suppression [38]. Notably, studies in non-small cell lung cancer (NSCLC) demonstrated that naive B cell infiltration correlates with favorable prognosis and tumor proliferation inhibition, while plasma cells exhibit stage-specific effects—partially suppressing early-stage NSCLC but promoting progression in advanced stages [29]. Although the underlying mechanisms remain unclear, these findings collectively suggest that dynamic changes in the tumor microenvironment may remodel plasma cell functions, indirectly supporting our observations.

To date, CD79A, CD20, SERPINA9, IGM, IGD, CD27, and TCL1A have been evaluated as markers for naïve B cells in different tumors [9, 29, 33, 39]. In our study, we selected TCL1A and CD72 as markers of naïve B cells. TCL1A is a member of the TCL1 family of AKT coactivators. The expression level of TCL1A is high in naïve B cells, decreases in germinal center B cells, and is absent in memory B cells [40]. CD72 is a member of the C-type lectin superfamily and is expressed on the surface of B cells from the pro-B through the mature B-cell stage [41]. We further identified 10 hub genes associated with highly infiltrating naive B cells, which were associated with T cells (*SELL*,* CD69*,* PTPRC*, and *CCR7*) and B cells (*CD22*,* PAX5*,* TNFRSF13C*,* CR2*,* BTK*,* PTPRC*, and *CD20*). This suggests that there are abundant T cells in the tissues with high infiltration of naive B cells, implicating a role of naïve B cells in formation of the TLS, which is an organized aggregate of immune cells in non-lymphoid tissues. SELL (L-Selectin) is a vascular cell adhesion molecule that is expressed by the leukocytes of myeloid-derived cells, naïve T cells, and some memory T cells [42]. In preclinical research, enhanced expression of SELL could improve the efficacy of CD8^+^ T cells in controlling both solid and disseminated tumor growth, whereas SELL-knockout T cells had no such effect [43]. Another study found that SELL can facilitate the rolling, sticking, and crawling behaviors of CD20^+^ lymphocytic leukemia cells on the endothelium of high endothelial venules [44]. Some studies have suggested that SELL can also serve as a marker for TLSs [45]. The presence of TLSs in tumors is correlated with a better prognosis and clinical outcomes upon immunotherapy [46]. In addition, our result showed that IgD + NPC patients not only has the better DMFS and OS than the IgD- patients, but also has higher CD20 + density. Although this is not direct evidence, it indirectly suggests that the patients with Naive B cells maybe have better prognostic value. Overall, these results suggest that naïve B cells may be involved in the TLS and serve as a prognostic marker for patients with NPC.

Our study has several limitations. First, our clinical data were obtained from a single center and the value of this result in the era of immunotherapy remains uncertain. Second, we distinguished the subtypes of B cells only using online data collected from other centers and did not validate these results using immunohistochemistry with an independent sample. Third, although we found that naïve B cells are closely related to the prognosis of NPC patients and may be closely related to the formation of TLS, the relationship between naïve B cells and TLS, and how they affect the prognosis of NPC patients through what mechanism still need more exploration in the future. In summary, our study showed that the density of infiltrating B cells was markedly associated with the prognosis of patients with NPC. The four common subtypes of B cells exhibited different degrees of infiltration in NPC and NNE tissues. Naïve B cells showed a high degree of infiltration into the NPC tissue, which was associated with a better prognosis compared to that of patients with low infiltration levels of naïve B cells. Several important immune pathways were identified to be associated with naïve B cells. Moreover, how to promote the infiltration of B cells, especially naïve B cells, is an important direction for follow-up research.

## Electronic supplementary material

Below is the link to the electronic supplementary material.

Supplementary Material 1

## Data Availability

The data that support the findings of this study are available within the paper and its Supplementary Information files. RNA sequencing data are from the GEO database. Patient information can be obtained from the corresponding author after publication with the approval of the Center’s Institutional Review Board.
