# Supplementary material for: Infiltrating B-cell subtypes and associated hub genes in nasopharyngeal carcinoma identified from integrated single-cell, bulk RNA-sequencing, and immunohistochemical data
Source: Hereditas. 2025 Mar 29;162:48. doi: 10.1186/s41065-025-00414-7 (PMC11954199; doi:10.1186/s41065-025-00414-7)
Supplement: Supplementary file 1 — Supplementary Material 1 [file 41065_2025_414_MOESM1_ESM.docx]

**Supplementary figures**

**Supplement figure 1**

**Supplement figure 1.** Representative B cell IHC staining images. (A) Low CD20+ B cells IHC staining images. (B) Number of low infiltrating CD20+ B cells in the tumor area were calculated by HALO software. (C) High CD20+ B cells IHC staining images. (D) Number of high infiltrating CD20+ B cells in the tumor area were calculated by HALO software.

**Supplement figure 2**

**Supplement figure 2.** Single-cell transcriptome datasets preprocessing. (A) The violin diagram is the number of genes in each cell, the number of mRNA molecules, and the proportion of mitochondrial genes. (B) To show the mitochondrial gene ratio and distribution of cell gene number under different mRNA molecule numbers. (C) The left figure is based on principal components 1 and 2 shows the cells on a flat surface, and the right figure shows the amount of interpretation for the first 50 principal components.

**Supplement figure 3**

**Supplement figure 3.** Clusters of immune cells in NPC and normal nasopharyngeal tissues. (A) t-SNE plot for 15 primary NPC tumors and one normal tissue. (B) t-SNE plot for immune cells from all cells, a total of 5 types of immune cell were identified. (C) t-SNE plot of immune cells in NNE tissues. (D) t-SNE plot of immune cells in NPC tissues. (E) the proportion of 5 immune cell types in 16 samples. (F) the expression of marker genes of B cells, T cells, CD4+T cells, CD8+T cells and dendritic cells in NNE tissues and NPC tissues has shown by heatmap.

**Supplement figure 4**

**Supplement figure 4.** The prognostic value of signatures in T cell activation and B cell receptor signaling pathway, respectively. (A) The prognostic value of signature in T cell activation. (B) The prognostic value of signature in B cell receptor signaling pathway.

**Supplement figure 5**

**Supplement figure 5.** Function enrichments in different B cell subtypes. (A, B) GO and KEGG enrichment of DEGs in switched memory B cells. (C, D) GO and KEGG enrichment of DEGs in plasma cells. (E) GO enrichment of DEGs in exhausted B cells. Color intensity is based on the adjust p value. (F) Metabolic pathway activity of B cell subtypes. (G) Distribution of metabolic pathway activity by B cell subtypes. (H) The top ten metabolic pathways are significantly enriched in all subtypes. (I) Hallmark pathway activity of B cell subtypes. (J) Distribution of Hallmark pathway activity in each B cell subtypes. (K) The top ten hallmark pathways that are significantly enriched in all subtypes.

**Supplement figure 6**

**Supplement figure 6.** cell-cell interaction network analysis. (A) cell-cell interaction network. (B) Heatmap shows the number of ligand-receptor pairs between different B cell subtypes. (C) Count of the top 40 interaction relationship pairs in each B cell subtype. (D) Bubble chart shows interaction pairs between B cell subtypes.

**Supplement figure 7**

**Supplement figure 7.** Prognosis, single-gene GSEA in pan-cancer of hub genes. (A-G) K-M curves of hub genes. (H) Results of single-gene GSEA enrichment of hub genes. (I) GSEA enrichment results and associated genes.

**Supplement figure 8**

**Supplement figure 8.** Clinical pathological features and expression level in pan-cancer of hub genes. (A) Average log2 (TPM+1) value (*p<0.05) of the hub genes in four pathological stages. (B) Differences expression of the hub genes between NPC samples and normal samples. (C, D) Expression levels of SELL and CD69 in pan-cancer and pair normal samples.

**Supplement figure 9**

**Supplement figure 9.** Representative IHC staining images of T-B spatial co-localization. (A) The representative IHC staining images of negative tertiary lymphoid structure. (B) The representative IHC staining images of non-mature tertiary lymphoid structure. (C) The representative IHC staining images of mature tertiary lymphoid structure.

**Supplementary materials and methods**

***Tissue samples and immunohistochemistry***

Tissue samples from 217 patients with NPC diagnosed at Jiangxi Cancer Hospital between January 2014 and December 2015 were included for analysis. All the patients have accepted radical radiotherapy. All patients with NPC were reclassified according to the TNM stage classification scheme of the 8th edition of the American Joint Committee on Cancer staging manual. This study was approved by the Hospital Review Board of Jiangxi Cancer Hospital (No.2023ky102), Jiangxi, China.

The samples were fixed in formaldehyde and processed using heat-mediated antigen retrieval in citrate buffer (pH 6). The samples were then blocked and incubated with rabbit polyclonal anti-CD20 antibody (1:250, ab78237, Abcam, Cambridge, MA USA) or rabbit monoclonal anti-IgD antibody (1:1000, ab124795, Abcam, Cambridge, MA USA) at 4°C overnight, followed by detection with Elivision^TM^ Plus Polymer HRP (mouse/rabbit) IHC Kit (Cat. KIT-9901; MXB Biotechnologies, China). HALO software was used to calculate the number of infiltrating CD20^+^ B cells in the tumor area[1]. The density of CD20^+^ B cells was calculated as the number of CD20^+^ B cells/tumor area (mm^2^). Patients were then divided into groups with high and low levels of infiltrating B cells according to the cut-off value of the infiltrating density of CD20^+^ B cells calculated with X-tile software.

***Public*** ***data sources***

Two NPC single-cell transcriptome datasets were downloaded from the Gene Expression Omnibus (GEO) database (<http://www.ncbi.nlm.nih.gov/geo/>): GSE150430 and GSE162025. The GSE150430 dataset contained 15 primary NPC tumor tissues and one normal nasopharyngeal epithelial (NNE) tissue, with a total of 48,584 cells (46,001 cells from NPC tissues and 2583 cells from the NNE tissue). The GSE162025 dataset contained 10 NPC tissues and paired peripheral blood samples, with a total of 17,6447 cells. Two bulk-RNA transcriptome datasets (GSE102349 and GSE34573) were also downloaded from the GEO database. The GSE102349 dataset contained mRNA expression levels and clinical data from 113 patients with NPC, whereas the GSE34573 dataset contained transcription data for 16 NPC tumor tissues and four NNE tissues. These publicly available datasets were analyzed with pre-existing ethics approval from the original studies.

***Identification of immune cell subsets***

Single-cell RNA-sequencing data were analyzed using the Seurat R package (version 3.1.5) [2]. Low-quality cells were removed based on a minimum gene number of 500, a maximum gene number of 7500, and a mitochondrial gene ratio of 2.5. Gene expression levels were normalized using the default parameters of the Seurat package. The FindVariableFeatures and ScaleData functions were used with the default parameters. Subsequently, principal component analysis (dimensions = 25) and FindClusters (resolution = 0.5) were performed to identify the cell clusters. Single cells were subjected to t-distributed stochastic neighbor embedding to identify five cell types (T cells, B cells, CD4^+^ T cells, CD8^+^T cells, and dendritic cells) [3]. Following standard Seurat procedures, four subsets of B cells (naïve B cells, switched memory B cells, exhausted B cells, and plasma cells) were identified from 19,154 B cells. These subsets were then annotated using data generated using the FindAllMarkers function in the Seurat package.

***Gene set enrichment of B-cell subsets***

Hallmark genes associated with B-cell subsets in NPC were evaluated using gene set enrichment analysis (GSEA) [4], single-sample gene set enrichment analysis (ssGSEA) [5], and gene set variation analyses (GSVA) [6]. GSEA classifies genes based on genomic annotation; ssGSEA is a special type of GSEA that is mainly used for single samples that cannot be subject to the classical GSEA approach, along with proposal of an implementation method. GSVA is a non-parametric, unsupervised method for estimating gene set enrichment variation according to the expression levels of samples from sequencing datasets. GSEA was conducted using the R package “clusterProfiler” [7], whereas ssGSEA and GSVA were conducted using the R package “GSVA.” Trajectory analysis for all B-cell subsets was performed using the R package “monocle.”

***Differentially expressed genes according to B-cell infiltration levels***

Differential gene expression analysis of samples with high and low levels of infiltrating B cells（based on the median value of infiltration）was conducted in the GSE102349 dataset using the R package “limma” [8] according to thresholds of p < 0.05 and |log fold change| > 1. The differentially expressed genes (DEGs) were visualized with heatmaps and volcano plots using the R package “pheatmap” and “EnhancedVolcano,” respectively.

***Pathway and functional enrichment analysis***

Pathway and functional enrichment analyses of the DEGs between high- and low-infiltrating B-cell groups were performed with Kyoko Encyclopedia of Genes and Genomes (KEGG) and Gene Ontology (GO) databases using the R package “ClusterProfiler” (version 4.0.5)[7]. A threshold of p < 0.05 was used to screen for significantly enriched pathways and functions.

***Cell–cell interactions and protein–protein interaction (PPI) network***

We used CellphoneDB[9] to identify interactive pairs with default parameters and to predict receptor–ligand interactions. In addition, we used the STRING database to conduct PPI analysis of the DEGs; the network was visualized using Cytoscape (version 3.9.1). The MCODE plugin was used to detect important co-modulation modules and the CytoHubba plugin was used to compute the hub nodes which were based on the intersecting DEGs between the bulk RNA-seq and scRNA-seq datasets.

***Statistical analysis***

Distant metastasis-free survival (DMFS) was defined as the time from randomization to the development of any distant metastasis or death. Overall survival (OS) was defined as the duration from the date of diagnosis to death from any cause or the last follow-up. Recurrence-free survival (RFS) was defined as the time elapsed between randomization and any recurrence (local, distant) or death for any reason. Progression free survival (PFS) was defined as duration from the date of diagnosis to disease progression or death from any cause. Pearson χ2 test or Fisher’s test was used to compare the basic clinical characteristics of patients in different groups. Kaplan–Meier survival analyses were used to estimate the DMFS, OS, RFS and PFS with log-rank test for comparison of survival curves. Multivariate analyses (MVA) with Cox proportional hazard methods were used to estimate the risk of death. The MVA models were constructed using a backward step. The criterion for the backward step is for significance at the 0.05 level before a variable could stay in the model, while parameters with a significance of greater than 0.10 were removed from the model. A bilateral P value <0.05 was statistically significant. All data in the study were analyzed using R (version 3.6.1) and SPSS (SPSS 26.0 IBM Corp, Armonk, NY).

**References：**

[1]	Zhao Z, Galiana G, Zillo C*, et al.* HALO: A software tool for real-time head alignment in the MR scanner Magn Reson Med 2022; 89: 1506-1513. <https://doi.org/10.1101/2022.03.23.485491>.

[2]	Satija R, Farrell JA, Gennert D*, et al.* Spatial reconstruction of single-cell gene expression data. Nat Biotechnol 2015; 33: 495-502. <https://doi.org/10.1038/nbt.3192>.

[3]	Kobak D and Berens P. The art of using t-SNE for single-cell transcriptomics. Nat Commun 2019; 10: 5416. <https://doi.org/10.1038/s41467-019-13056-x>.

[4]	Subramanian A, Tamayo P, Mootha VK*, et al.* Gene set enrichment analysis: a knowledge-based approach for interpreting genome-wide expression profiles Proc Natl Acad Sci U S A 2005 Oct 25; 102: 15545-15550. <https://doi.org/10.1073/pnas.0506580102>

[5]	Barbie DA, Tamayo P, Boehm JS*, et al.* Systematic RNA interference reveals that oncogenic KRAS-driven cancers require TBK1. Nature 2009; 462: 108-112. <https://doi.org/10.1038/nature08460>.

[6]	Hänzelmann S, Castelo R and Guinney J. GSVA: gene set variation analysis for microarray and RNA-seq data BMC Bioinformatics 2013 Jan 16; 16: 7. <https://doi.org/10.1186/1471-2105-14-7>.

[7]	Yu G, Wang LG, Han Y*, et al.* clusterProfiler: an R package for comparing biological themes among gene clusters. OMICS 2012; 16: 284-287. <https://doi.org/10.1089/omi.2011.0118>.

[8]	Ritchie ME, Phipson B, Wu D*, et al.* limma powers differential expression analyses for RNA-sequencing and microarray studies. Nucleic Acids Res 2015; 43: e47. <https://doi.org/10.1093/nar/gkv007>.

[9]	Efremova M, Vento-Tormo M, Teichmann SA*, et al.* CellPhoneDB: inferring cell-cell communication from combined expression of multi-subunit ligand-receptor complexes. Nat Protoc 2020; 15: 1484-1506. <https://doi.org/10.1038/s41596-020-0292-x>.

**Supplementary Tables**

**Supplementary Table 1.** Demographic and clinical characteristics of NPC patients with high- and low infiltration density of CD20+ B cells in our center (n = 217).

| **Characters** | **Classification** | **CD20+ B cells infiltration density level** | | **p-value** |
| --- | --- | --- | --- | --- |
|  |  | **High/(n=178)** | **Low/(n=39)** |  |
| Gender | Female | 43 | 13 | 0.236 |
|  | Male | 135 | 26 |  |
| Age (median [IQR]) |  | 48[38.25,56] | 45[38,56] | 0.898 |
| Pathological Type | Nonkeratinizing carcinoma | 164 | 37 | 0.065 |
|  | Keratinizing squamous cell carcinoma | 14 | 2 |  |
| T classification | T1 | 49 | 7 | 0.32 |
|  | T2 | 30 | 4 |  |
|  | T3 | 41 | 11 |  |
|  | T4 | 58 | 17 |  |
| N classification | N0 | 19 | 4 | 0.899 |
|  | N1 | 101 | 21 |  |
|  | N2 | 46 | 10 |  |
|  | N3 | 12 | 4 |  |
| Clinical stage | Stage I | 7 | 2 | 0.517 |
|  | Stage II | 41 | 5 |  |
|  | Stage III | 63 | 14 |  |
|  | Stage IV | 67 | 18 |  |
| Infiltrating density of CD20 + B cells (mdian [IQR]) (number/mm2) |  | 2707.8  [1478.9, 4520.6] | 366.3  [186.4, 487.1] | <0.001 |
| EBV-DNA (Copies/ml) | <500 | 136 | 30 | 0.945 |
|  | ≥500 | 42 | 9 | 0.945 |

| Characteristics | DMFS-Multivariate analysis | | OS-Multivariate analysis | | PFS-Multivariate analysis | | RFS-Multivariate analysis | |
| --- | --- | --- | --- | --- | --- | --- | --- | --- |
|  | HR (95% CI) | p-value | HR (95% CI) | p-value | HR (95% CI) | p-value | HR (95% CI) | p-value |
| Gender（Male vs. Female） | 1.988(0.822-4.81) | 0.127 | 2.02(0.846-4.825) | 0.113 | 1.977(0.917-4.26) | 0.082 | 1.599(0.452-5.658) | 0.467 |
| Age (>60 vs. <=60) | 2.907(1.443-5.855) | 0.003 | 3.703(1.9670-6.968) | <0.001 | 2.369(1.248-4.498) | 0.008 | 4.601(1.697-12.479) | 0.003 |
| T classification (T3-4 vs. T1-2) | 1.276(0.639-2.547) | 0.49 | 1.364(0.711-2.614) | 0.35 | 0.936(0.51-1.717) | 0.83 | 0.818(0.31-2.158) | 0.685 |
| N classification (N2-3vs. N0-1) | 1.454(0.769-2.749) | 0.25 | 3.755(0.902-15.639) | 0.069 | 1.554(0.887-2.725) | 0.124 | 2.791(1.097-7.097) | 0.031 |
| Density of CD20+ B cells (Low vs. High) | 2.819(1.446-5.495) | 0.002 | 2.102(1.08-4.09) | 0.029 | 2.979(1.635-5.428) | <0.001 | 1.67(0.581-4.804) | 0.341 |
| EBV-DNA (>500 vs. <=500) | 1.11(0.539-2.289) | 0.777 | 1.037(0.552-1.948) | 0.91 | 0.817(0.415-1.61) | 0.56 | 0.136(0.018-1.045) | 0.055 |

**Supplementary Table 2.** Multivariate analyses of distant metastasis-free survival (DMFS), overall survival (OS), progression-free survival (PFS), and recurrence-free survival (RFS) of patients with nasopharyngeal carcinoma according to the density of tumor-infiltrating CD20^+^ B cells (n = 217).

**Supplementary Table 3.** The genes of signatures in T cell activation and B cell receptor signaling pathway, respectively.

| **T cell activation pathway** | **B cell receptor signaling pathway** |
| --- | --- |
| CD55, CD83, FOXP1, PTPRC, HLA-DRB1, HLA-DMB, RHOH, GRB2, IL4R, LY9, LYN, TNFRSF13C, CCR6, DOCK8, APBB1IP, BTLA, TGFBR2, NFKBIZ, PAK2, CCR7, CD1D, MALT1, ZBTB1, ICOSLG, CHD7, CCDC88B, HLA-DOA, PIK3R1, NFKBID, BTN2A2, AP3B1 | MAPK3, HRAS, NFATC4, CD79B, VAV1, MAPK8 , MAP2K1, RAF1, BTK, ELK1, PLCG1, CALM1, NFATC2, SYK, RAC1, SHC1, PPP3CA, NFATC3, GRB2, PRKCB, CALM2, MAPK14, LYN, PPP3CB, CALM3, PRKCA, NFATC1, CD79A, MAP3K1, JUN, SOS1, FOS, PPP3CC |
